# Comprehensive Medial Knee Four‐Arm Reconstruction to Address Complex Instability With Hamstring Autograft

**DOI:** 10.1002/atn2.70067

**Published:** 2026-08-03

**Authors:** Konrad Malinowski, Kamil Możdżeń, Michalina Bawor‐Mostowa, Przemysław Pękala, Andrew A. Amis, Marcin Mostowy

**Affiliations:** ^1^ Artromedical Orthopedic Clinic Bełchatów Poland; ^2^ Department of Anatomy Jagiellonian University Medical College, International Evidence‐Based Anatomy Working Group Kraków Poland; ^3^ Department of Orthopedics and Traumathology Jagiellonian University Collegium Medicum Kraków Poland; ^4^ Faculty of Medicine and Health Sciences Andrzej Frycz Modrzewski Kraków University Kraków Poland; ^5^ Lesser Poland Orthopedic and Rehabilitation Hospital Kraków Poland; ^6^ Mechanical Engineering Department Biomechanics Group, Imperial College London London UK; ^7^ Orthopedic and Trauma Department Veterans Memorial Teaching Hospital in Lodz, Medical University of Lodz Lodz Poland

## Abstract

Medial knee ligament injuries are increasingly recognized as complex, multiplanar lesions, resulting not only in simple medial instability but also anteromedial rotatory instability, anteromedial instability, posteromedial instability, posteromedial rotatory instability, and post‐traumatic medial asymmetric hyperextension. The described technique extensively addresses all 6 above‐described types of instability. It is based on 4 reconstruction arms with independent fixation. Two arms are created from a pedunculated semitendinosus autograft and 2 arms from a free gracilis autograft. The technique can reconstruct the medial ligamentous complex from the anteromedial aspect (anteromedial capsule/anterior oblique ligament/deep medial collateral ligament), through the superficial medial collateral ligament and posterior oblique ligament, up to the posteromedial capsule, in response to the pathology identified.

## Graphical abstract

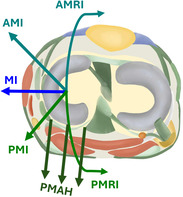

**VIDEO 1:** We present the comprehensive medial knee reconstruction technique addressing all types of medial instability: not only simple medial instability but also anteromedial rotatory instability, anteromedial instability, posteromedial instability, posteromedial rotatory instability, and post‐traumatic medial asymmetric hyperextension (Figure 1). Right knee is presented, patient supine. A medial hockey stick incision is made. The sartorial fascia is exposed and incised between the semitendinosus tendon (ST‐T) and gracilis tendon (G‐T). ST‐T is harvested proximally using an open tendon harvester, leaving its distal insertion intact, creating a pedunculated graft. It will serve as the anteromedial and medial arm of the technique. The G‐T is harvested as a free graft. Next, the sartorial fascia is incised with scissors up to the medial epicondyle. The K‐wire is placed 5 mm proximal to the anatomic attachment of superficial medial collateral ligament (sMCL) and isometry of ST‐T is assessed. ST‐T is folded over the GraftMax device (Conmed, Warsaw, Poland) and sutured with a Krackow stitch along a 2 cm section using Ethibond Excel nonabsorbable thread (Ethicon, Johnson and Johnson, Warsaw, Poland). The G‐T is folded and sutured in the same manner but positioned backward in relation to the ST‐T. The free ends of the G‐T are sutured with a standard Krackow stitch. Next, a 4.5 mm femoral tunnel is drilled bicortically for GraftMax (Conmed, Warsaw, Poland) passage and a 25 to 30 mm deep socket is drilled with a diameter of ST‐T and G‐T diameter together. The GraftMax (Conmed, Warsaw, Poland) is secured in a standard manner and used to pull the grafts into the femoral socket. Afterward, isometric point for distal sMCL tunnel is to be found. It is located approximately 6 to 7 cm below the joint space as close to posteromedial cortex of the tibia as possible. Tibial tunnel for sMCL is drilled bicortically with 4.5 mm drill and unicortically with a drill 1 to 1.5 mm larger that the measured graft diameter. The graft is double secured: with a Genesys bioabsorbable screw (Conmed, Warsaw, Poland) and with tibial button. As a next step, we palpate for the direct arm of semimembranosus and incise its bursa just above its attachment. Within the bursa, tibial tunnels for the reconstruction of posterior oblique ligament and for the reconstruction of the posteromedial capsule will be drilled. Two K‐wires are inserted in the bursa, aiming at the anterolateral part of the tibia. The first one will guide POL tibial tunnel drilling. The second, intended to guide posteromedial capsule tibial tunnel, is more lateral and distal but still inside the bursa. After measuring the diameters of both arms of the G‐T graft, 2 separate 25 to 30 mm tunnels are drilled. Next, both arms of the G‐T graft are pulled into the tibial tunnels, and each is stabilized separately, close to full extension, using a Genesys (Conmed, Warsaw, Poland) bioabsorbable screws. They will serve as the posteromedial and posterior arm of the technique. The complete technique addresses 6 types of medial knee instability in an extensive manner. It reconstructs the medial ligamentous complex from the anteromedial aspect, through the sMCL and posterior oblique ligament, to the posteromedial capsule. Video content can be viewed at https://doi.org/10.1002/atn2.70067.

Classically, medial knee ligament injuries were mainly treated conservatively.^1^ Throughout the years, various techniques for reconstruction of the superficial medial collateral ligament (sMCL) have been described.^2^, ^3^, ^4^, ^5^ Recently, increasing evidence has resulted in gradual recognition of the complexity and importance of the medial knee ligamentous apparatus.^6^, ^7^, ^8^, ^9^, ^10^, ^11^ This has resulted in the publication of surgical techniques in which the sMCL was reconstructed along with the posterior oblique ligament (POL), posteromedial capsule (PMC), deep MCL (dMCL), or anteromedial reinforcement.^2^, ^4^, ^12^ A trend can be seen toward more comprehensive medial knee ligamentous complex reconstruction, either by combined reconstruction of multiple ligaments or by the usage of flatter, wider grafts.^13^ Increasingly extensive medial knee ligamentous reconstructions aim to treat not only medial instability (MI) but also rotational instabilities.^14^, ^15^

In this technical note, we describe the technique designed to address not only the MI, but also anteromedial rotatory instability (AMRI), anteromedial instability (AMI), posteromedial instability (PMI), posteromedial rotatory instability (PMRI) and post‐traumatic medial asymmetric hyperextension (PMAH) according to clinical indications (Figure 1, Video 1).

**FIGURE 1:**
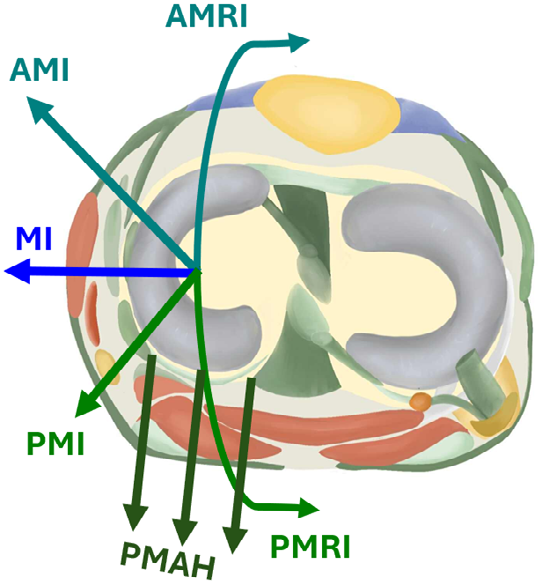
Six types of instability addressed by the technique. (AMI, anteromedial instability; AMRI, anteromedial rotatory instability; MI, medial instability; PMAH, post‐traumatic medial asymmetric hyperextension; PMI, posteromedial instability; PMRI, posteromedial rotatory instability.)

## SURGICAL TECHNIQUE

### Patient Positioning and Preparation

The patient lies supine with tourniquet applied. First, arthroscopic confirmation of preoperative diagnosis and treatment of coexisting intra‐articular pathologies is performed. The exception is anterior/posterior cruciate ligament reconstruction, which is performed after medial reconstruction.

### Technique

**FIGURE 2:**
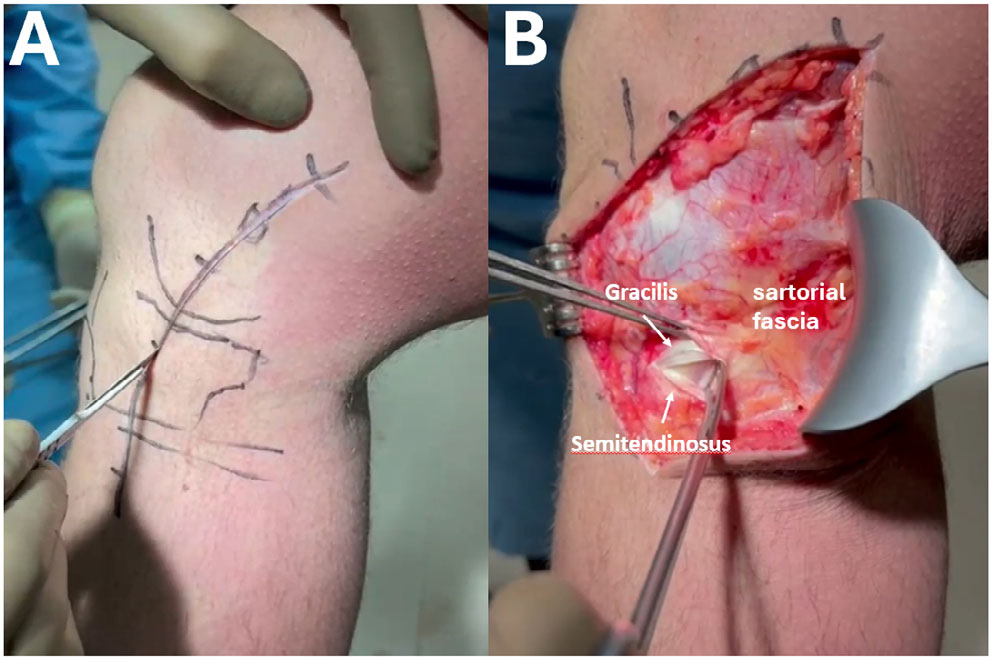
Right knee, patient supine. (A) A hockey stick incision is made, extending from 1.5 cm proximal to the medial epicondyle to 2 cm medial and distal the tibial tuberosity. (B) The sartorial fascia is exposed and incised between the semitendinosus ST‐T and G‐T. (G‐T, gracilis tendon; ST‐T, semitendinosus tendon.)

**FIGURE 3:**
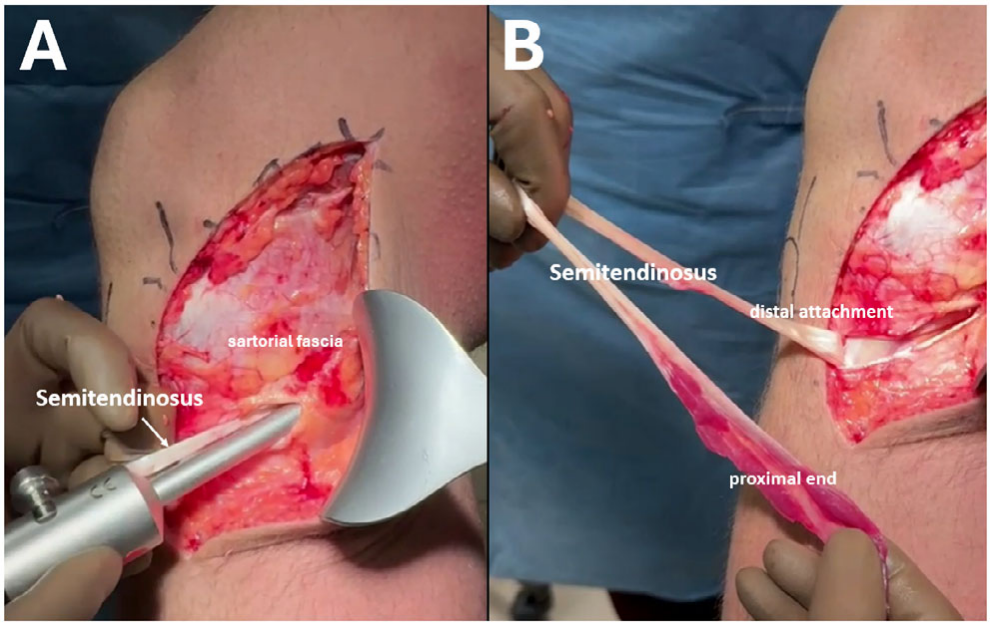
Right knee, patient supine. (A) Semitendinosus harvested proximally using an open tendon harvester (Conmed, Warsaw, Poland), leaving the distal attachment intact (B).

**FIGURE 4:**
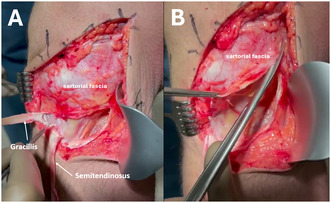
Right knee, patient supine. (A) The distal attachment of G‐T is cut from the tibia after harvesting. (B) The sartorial fascia incised with scissors up to the ME. (G‐T, gracilis tendon; ME, medial epicondyle.)

A hockey stick incision is made from 1.5 cm proximal to the medial epicondyle to 2 cm medial and distal to the tibial tuberosity (Figure 2A, Video 1). The sartorial fascia is exposed and incised between the semitendinosus (ST‐T) and gracilis (G‐T) tendons (Figure 2B). The ST‐T is harvested proximally using an open tendon harvester (Conmed, Warsaw, Poland), leaving its distal insertion intact (Figure 3, Video 1). The G‐T is harvested as a free graft (Figure 4A, Video 1). Next, the sartorial fascia is incised with scissors up to the medial epicondyle (Figure 4B).

**FIGURE 5:**
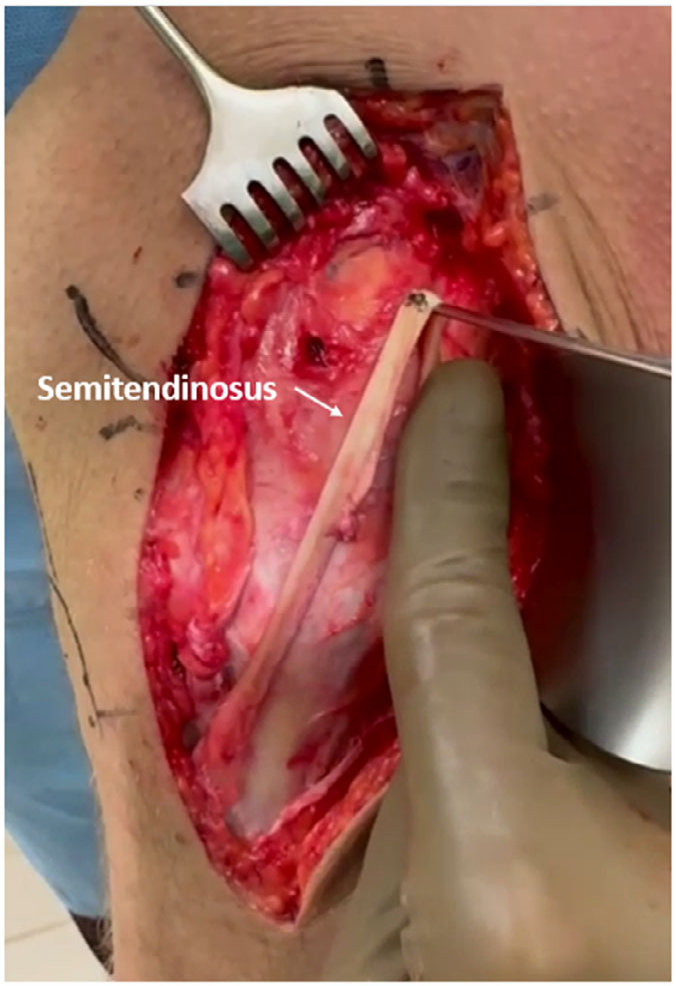
Right knee, patient supine. Assessment of isometry of the femoral tunnel using the proximally harvested semitendinosus tendon.

To find the common femoral tunnel location, a 1.6 mm K‐wire is placed 5 mm proximal to the anatomic sMCL attachment. Then, the free proximal end of the ST‐T graft is wrapped around the K‐wire and tensioned manually, and isometry is assessed (Figure 5, Video 1). The final femoral isometric point for the technique is usually located approximately 10 mm proximally from the medial epicondyle.

**FIGURE 6:**
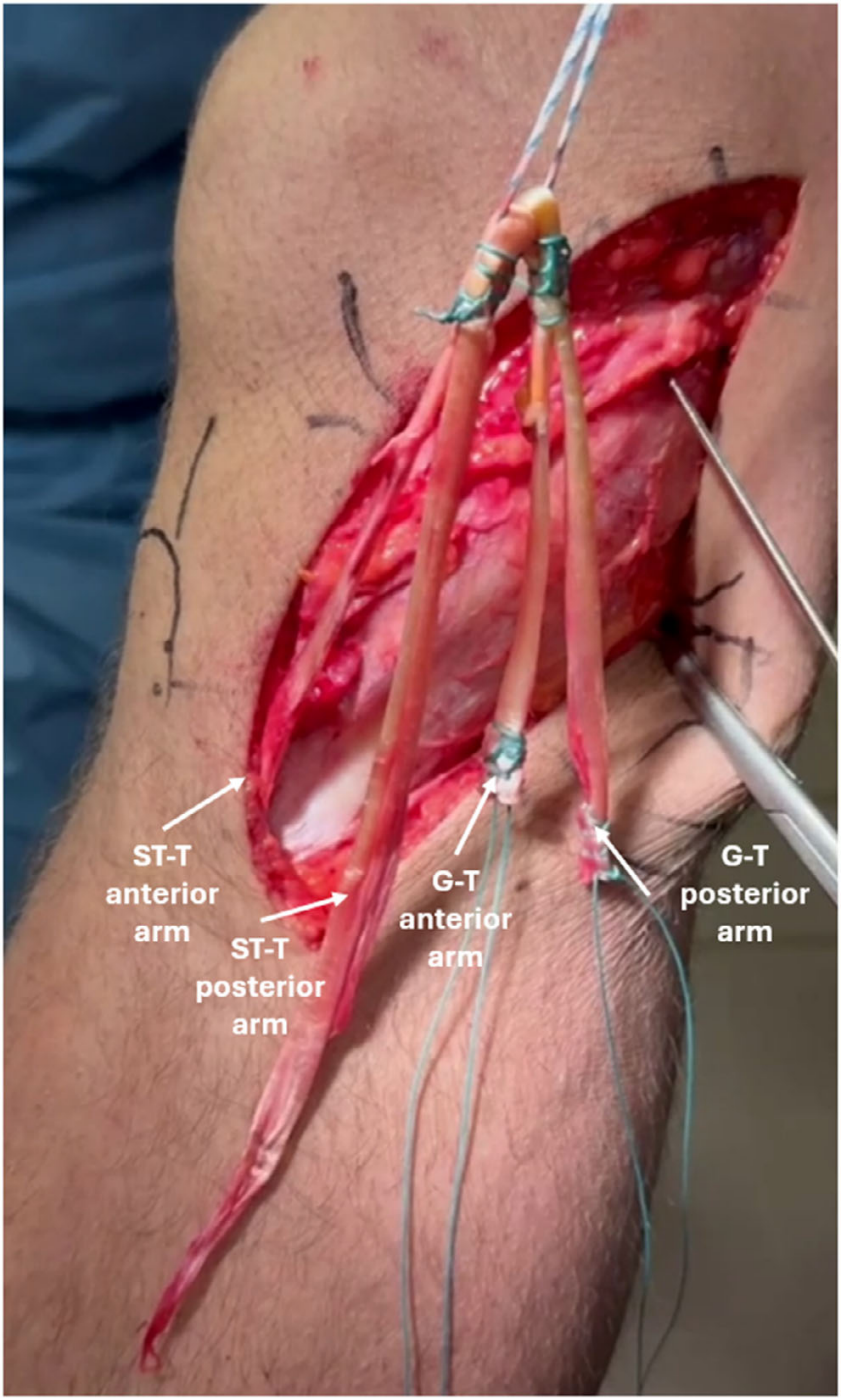
Right knee, patient supine. ST‐T and G‐T grafts folded and sutured over the GraftMax device (Conmed, Warsaw, Poland). From the left side: pedunculated anterior arm of ST‐T, free posterior arm of ST‐T, free anterior arm of G‐T, and free posterior arm of G‐T. (G‐T, gracilis tendon; ST‐T, semitendinosus tendon.)

After the location of the femoral tunnel is defined, ST‐T is folded over the GraftMax device (Conmed, Warsaw, Poland) and sutured with a Krackow stitch along a 2 cm section using nonabsorbable thread no. 2 Ethibond Excel (Ethicon, Johnson and Johnson, Warsaw, Poland). The G‐T is also folded over the GraftMax device (Conmed, Warsaw, Poland) on the same loop and sutured analogously but positioned backward in relation to the ST‐T. The free ends of the G‐T are sutured with a standard Krackow stitch using the same thread (Figure 6, Video 1).

**FIGURE 7:**
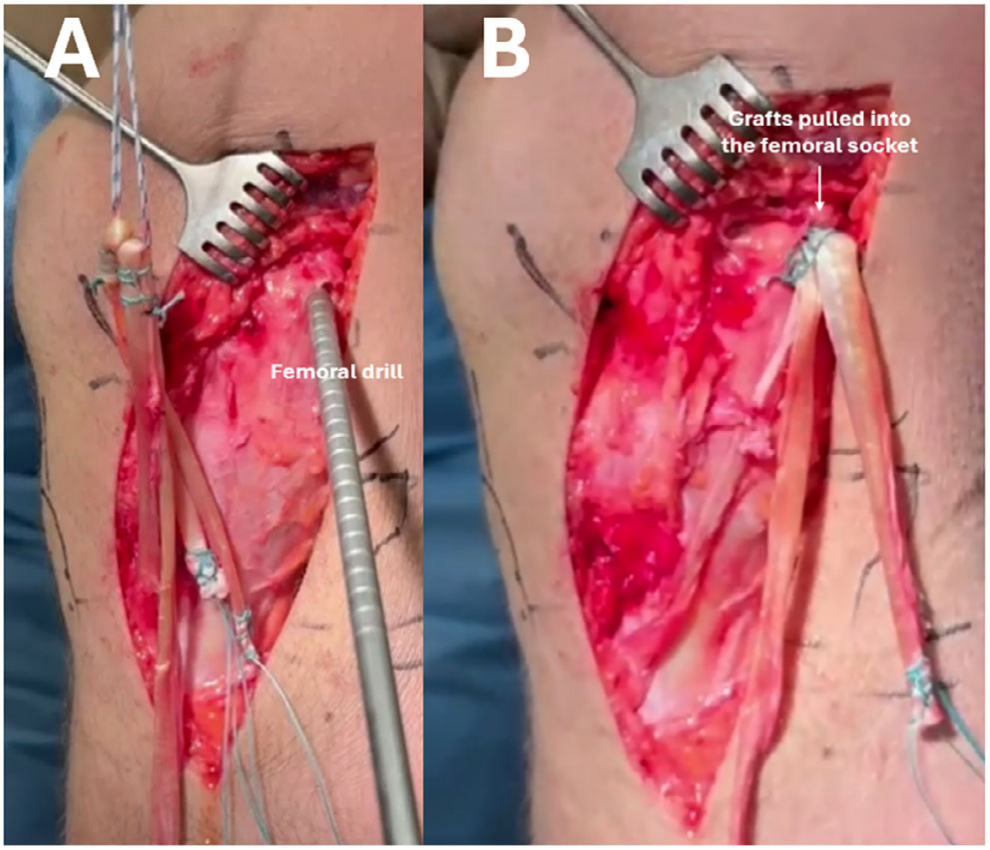
Right knee, patient supine. (A) Femoral socket is drilled. (B) Grafts pulled into the femoral socket.

Next, using the K wire as a guide, a 5 mm femoral tunnel is drilled bicortically for GraftMax (Conmed, Warsaw, Poland) passage. Next, a 25 to 30 mm deep socket is drilled with a diameter consistent with measurement of the diameter of the ST‐T and G‐T grafts together (Figure 7A, Video 1). The femoral tunnel is directed in the anterior‐proximal direction to avoid convergence with the intercondylar notch or possible posterior cruciate ligament reconstruction femoral tunnels. The GraftMax (Conmed, Warsaw, Poland) is secured, and the grafts are pulled into the femoral socket (Figure 7B).

**FIGURE 8:**
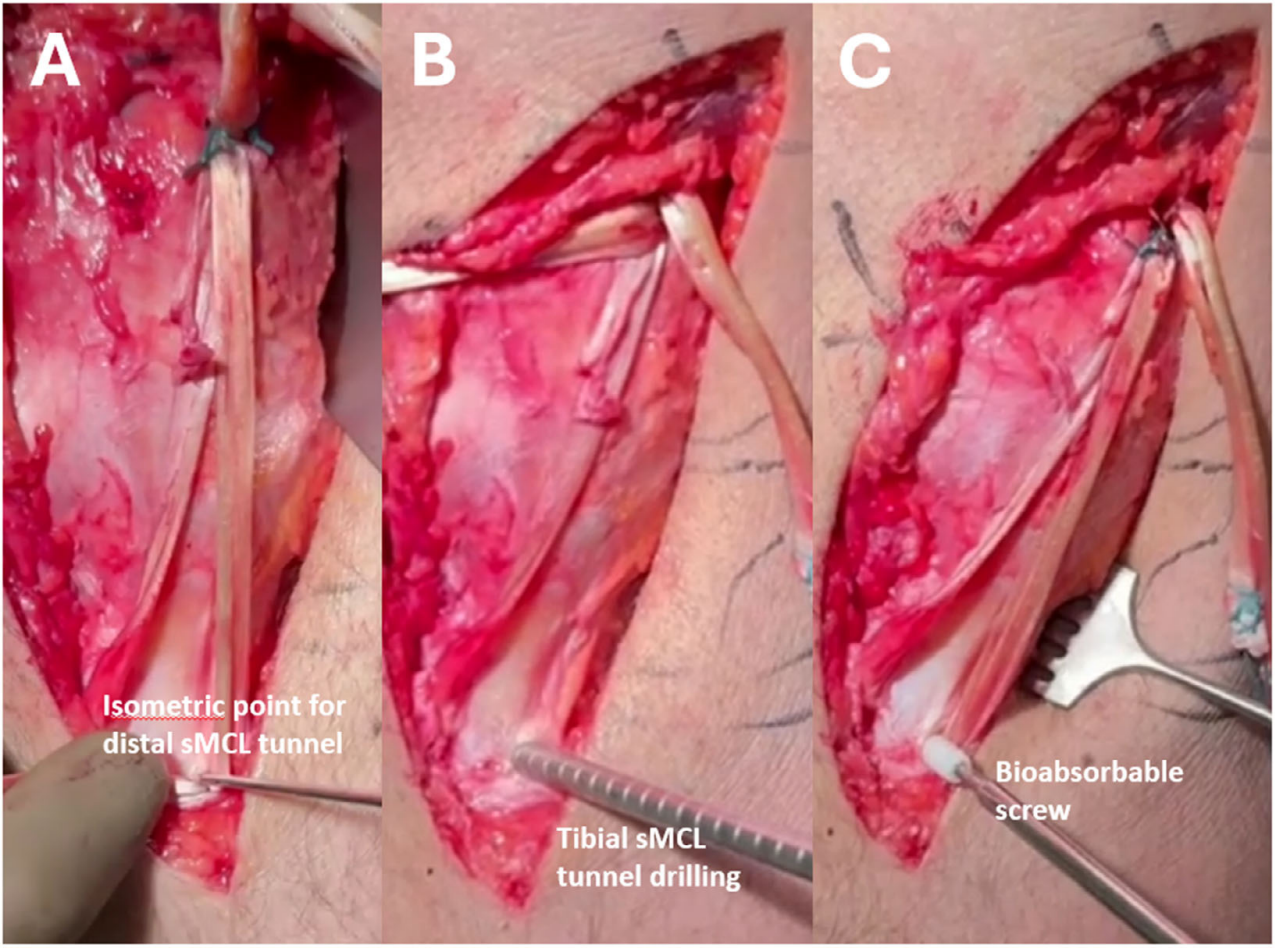
Right knee, patient supine. (A) Isometric point for distal sMCL tunnel. (B) Drilling of distal sMCL tunnel. (C) Fixation. (sMCL, superficial medial collateral ligament.)

Afterward, the isometric point for the distal sMCL tunnel is found. It is located approximately 6 to 7 cm below the joint space as close to posteromedial cortex of the tibia as possible, which corresponds to the anatomic sMCL attachment (Figure 8A, Video 1). The free end of the ST‐T graft is cut and sutured so that it reaches 20 mm beyond the tibial isometric point. The tibial tunnel for the sMCL is drilled bicortically with 4.5 mm drill and unicortically with a drill 1 to 1.5 mm larger than the measured graft diameter (Figure 8B). The ST‐T graft is double secured in this tunnel while the knee is in neutral rotation and approximately 10° of flexion: with a Genesys bioabsorbable screw (Conmed, Warsaw, Poland), 1 mm smaller than the graft diameter and with an Infinity Tibial Button (Conmed, Warsaw Poland) on the anterolateral side of the tibia (Figure 8C).

**FIGURE 9:**
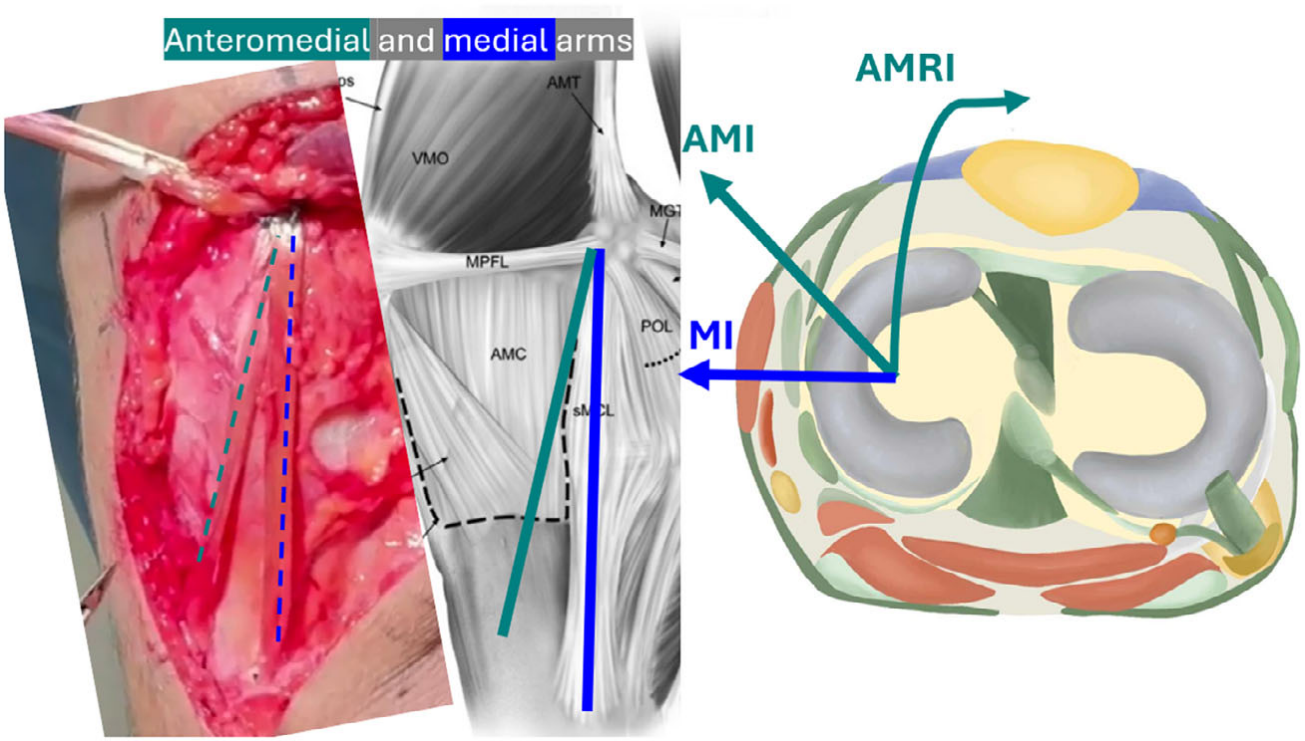
Right knee, patient supine. Anteromedial and medial arms of technique. Knee anatomical drawing reproduced from Vap et al. under Creative Commons license.^11^ (AMC, anteromedial capsule; AMI, anteromedial instability; AMRI, anteromedial rotatory instability; AMT, adductor magnus tendon; MGT, medial gastrocnemius tendon; MI, medial instability; MPFL, medial patellofemoral ligament; POL, posterior oblique ligament; sMCL, superficial medial collateral ligament; VMO, vastus medialis oblique.)

At this point, the anteromedial and medial arms of the technique have been created, stabilizing the knee against AMI, AMRI, and MI (Figure 9, Video 1). The anteromedial arm restores the stability of the anteromedial aspect of the knee: anteromedial capsule/anterior oblique ligament/dMCL. The medial arm reconstructs the sMCL (Figure 9). Up to this point, the technique is similar to that previously published by Malinowski et al.^2^

**FIGURE 10:**
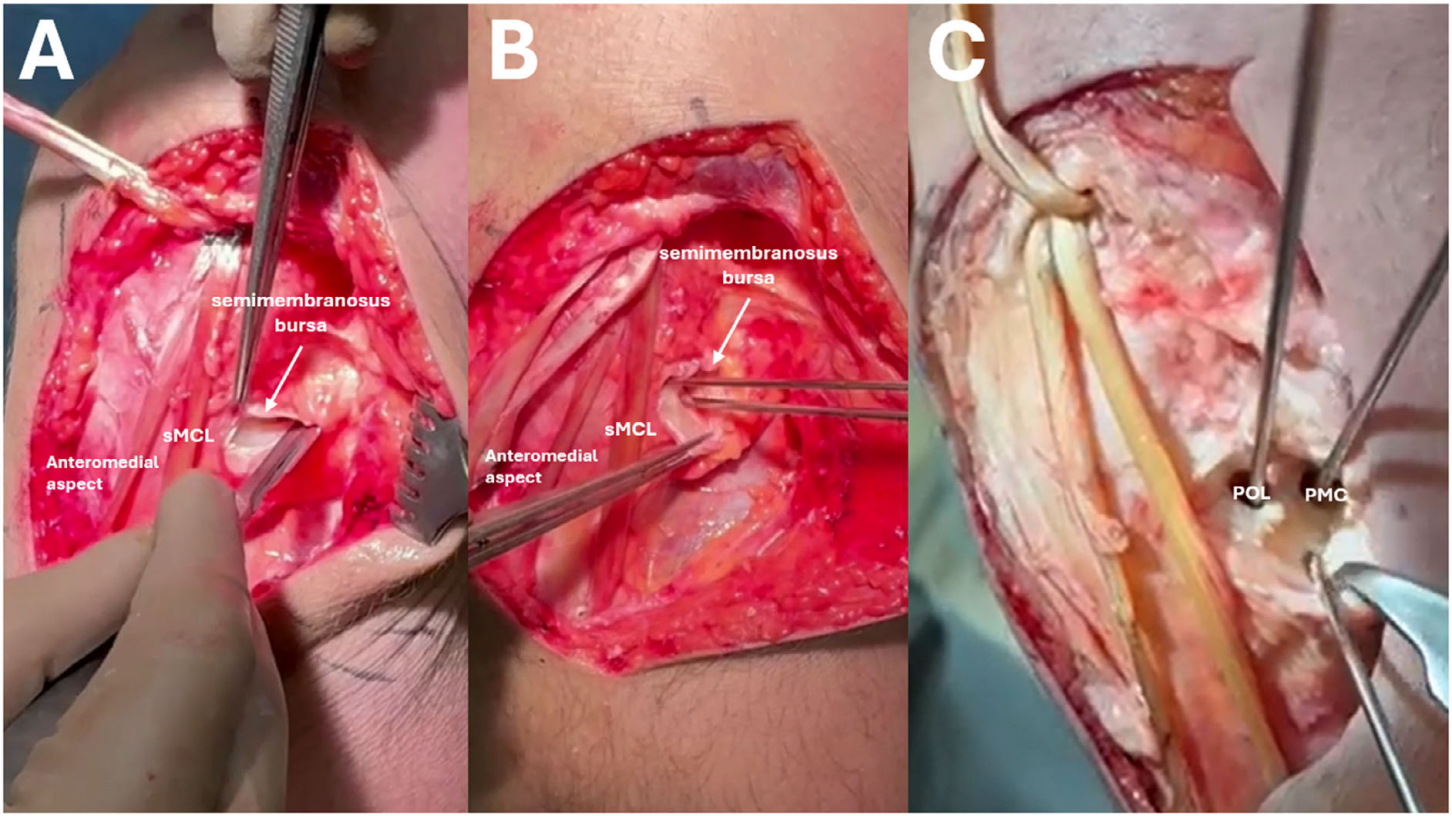
Right knee, patient supine. (A) The semimembranosus bursa incised above the semimembranosus tendon. (B) Two K‐wires inserted in the semimembranosus bursa, aiming at the anterolateral part of the tibia, approximately 2 cm distally and 1 cm laterally to the Gerdy's tubercle. Those will guide drilling of 2 tunnels: the first, located approximately 1 cm below the joint space, is responsible for POL reconstruction, while the second, located slightly distal and lateral, is intended for PMC reconstruction. (C) Tibial tunnels for POL and PMC reconstruction drilled within the semimembranosus bursa. (PMC, posteromedial capsule; POL, posterior oblique ligament; sMCL, superficial medial collateral ligament.)

As a next step, we palpate for the direct arm of the semimembranosus and incise its bursa just above it (Figure 10A, Video 1). Two K‐wires are inserted aiming toward the anterolateral aspect of the tibia, approximately 20 mm distally and 10 mm laterally to Gerdy's tubercle. The first, for POL tibial tunnel drilling, is located approximately 10 mm below the joint space, slightly anterior to the direct arm of semimembranosus, within the semimembranosus bursa.^4^ The second, intended to guide drilling of the tibial tunnel for PMC reconstruction, is usually located 10 to 20 mm distally and laterally from the first K‐wire. Its exact location is dependent on the size of semimembranosus bursa (Figure 10B). After measuring the diameters of the G‐T graft arms, 2 separate 25 to 30 mm tunnels are drilled to the measured graft diameter (Figure 10C).

Note that, although the anteromedial and medial (ST‐T) arms of the technique are isometric, the posteromedial and posterior arms are not. This is deliberate because this part of the medial ligamentous complex slackens with knee flexion.^13^ Therefore, the posteromedial and posterior arms are fixed near full extension to avoid overconstraint.

**FIGURE 11:**
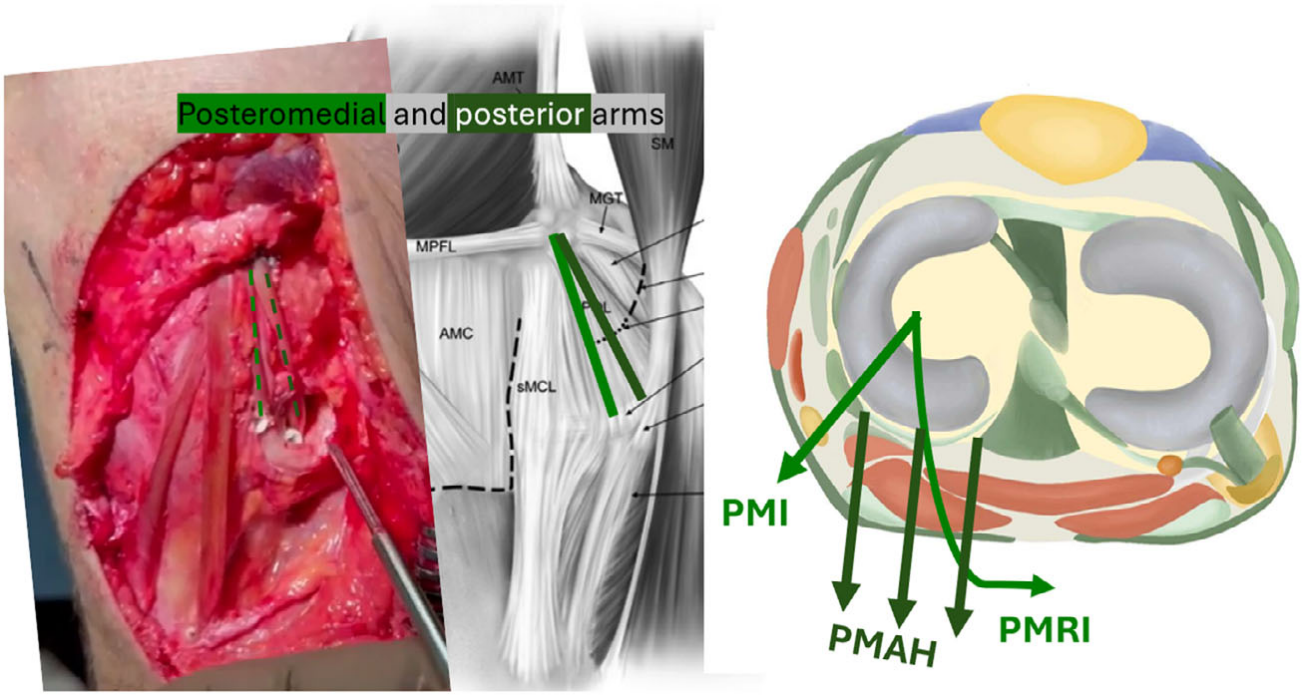
Right knee, patient supine. Posteromedial and posterior arms of technique. Knee anatomical drawing reproduced from Vap et al. under Creative Commons license.^11^ (AMC, anteromedial capsule; AMT, adductor magnus tendon; MGT, medial gastrocnemius tendon; MPFL, medial patellofemoral ligament; PMAH, post‐traumatic medial asymmetric hyperextension; PMI, posteromedial instability; PMRI, posteromedial rotatory instability; SM, semimebranosus muscle; sMCL, superficial medial collateral ligament.)

Next, the anterior and posterior arms of the G‐T graft are inserted into the tibial tunnels and fixed separately, close to full extension, using Genesys (Conmed, Warsaw, Poland) bioabsorbable screws with their diameters matching the drilled tunnels. The posteromedial arm reconstructs the POL, while the posterior arm reconstructs the PMC. These posteromedial and posterior arms of the technique stabilize the knee against PMI, PMRI, and PMAH (Figure 11, Video 1).

**FIGURE 12:**
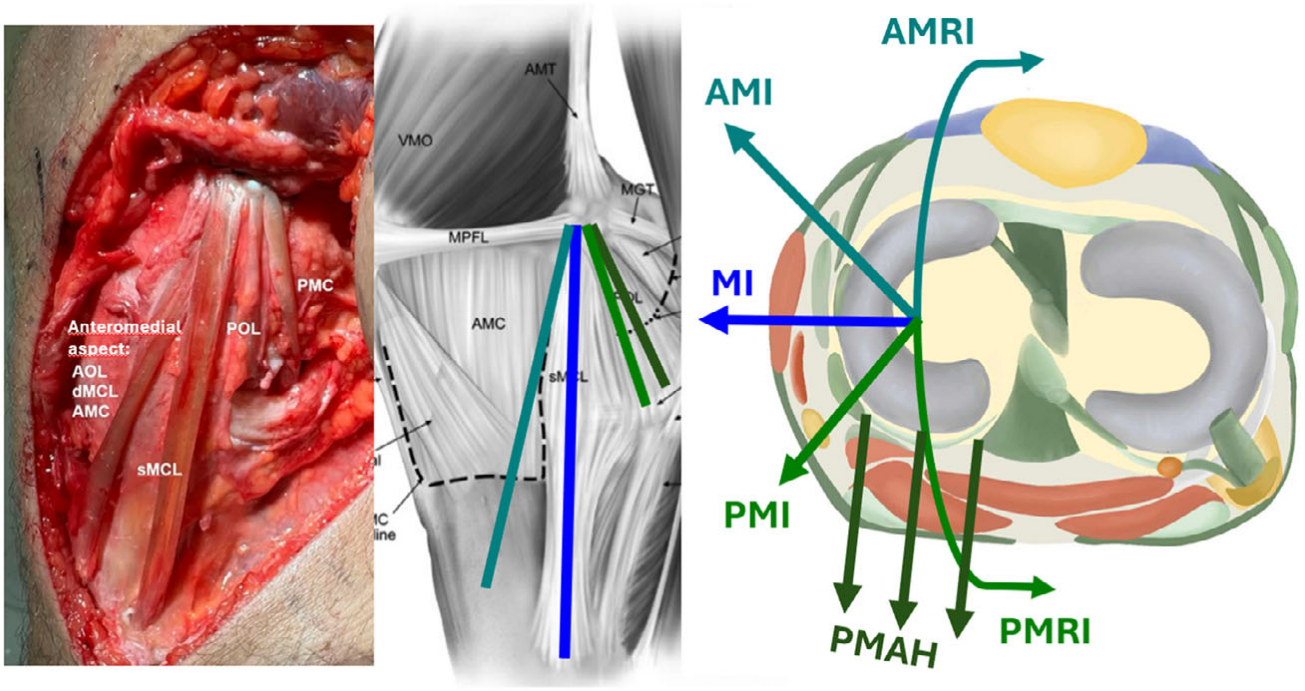
Right knee, patient supine. The complete technique. Knee anatomical drawing reproduced from Vap et al. under Creative Commons license.^11^ (AMC, anteromedial capsule; AMI, anteromedial instability; AMRI, anteromedial rotatory instability; AMT, adductor magnus tendon; AOL, anterior oblique ligament; dMCL, deep medial collateral ligament; MGT, medial gastrocnemius tendon; MI, medial instability; MPFL, medial patellofemoral ligament; PMAH, post‐traumatic medial asymmetric hyperextension; PMC, posteromedial capsule; PMI, posteromedial instability; PMRI, posteromedial rotatory instability; POL, posterior oblique ligament; sMCL, superficial medial collateral ligament; VMO, vastus medialis oblique.)

The complete technique addresses 6 types of medial knee instability: AMRI, AMI, MI, PMI, PMRI, and PMAH. It reconstructs the medial ligamentous complex from the anteromedial aspect (anteromedial capsule/anterior oblique ligament/dMCL), through the sMCL and POL, to the PMC (Figure 12, Video 1). Finally, the sartorial fascia, subcutaneous tissue, and skin are closed. Pearls and pitfalls of the technique are summarized in Table 1, and advantages and disadvantages are discussed in Table 2.

**TABLE 1:** Pearls and Pitfalls

1. It is important to ensure that the GraftMax loop can move smoothly relative to the grafts and is not blocked by the sutures.
2. The distal sMCL tunnel is drilled within the cortical, hard part of the tibia. Therefore, double fixation is used to enable the usage of small diameter bioabsorbable screw to avoid cutting the graft.
3. Anteromedial and medial arms of the technique are isometric, while posteromedial and posterior arms are not. This is deliberate, due to biomechanical properties of this part of the medial ligamentous complex. Therefore, the posteromedial and posterior arms are fixed near full extension.
4. While pulling the grafts into the femoral tunnel, it is crucial to pay attention that the designed arms remain in respective order. The surgeon must avoid rotation of the grafts.
5. Improper identification of isometric femoral point will result in residual instability.
6. Tibial tunnels for POL and PMC have to be aimed approximately 2 cm distally and 1 cm laterally to Gerdy's tubercle to avoid convergence with posterolateral corner reconstruction tibial tunnel and common peroneal nerve injury.
7. It is possible to perform the surgery using 3 mini‐open incisions.

PMC, posteromedial capsule; POL, posterior oblique ligament; sMCL, superficial medial collateral ligament.

**TABLE 2:** Advantages and Disadvantages of the Technique

Advantages	Disadvantages
1) Addresses 6 types of medial knee instability in an extensive manner	1) More hardware than in isolated sMCL reconstruction
2) Separate stabilization of arms allows to restore distinct isometry/anisometry patterns	2) More extensive approach than in isolated sMCL reconstruction
3) Based on previous surgical techniques	3) Both hamstrings are needed for reconstruction
4) No more surgical instruments than in the ACL hamstring‐based reconstructions are necessary	
5) Possible personalization of reconstruction	

ACL, anterior cruciate ligament; sMCL, superficial medial collateral ligament.

### Rehabilitation Protocol

The patients ambulates with crutches for about 6 weeks. Passive range of motion is from 0° to 90° from the first postoperative day. For the first 6 weeks, the patient wears a simple orthosis to immobilize the limb in extension for walking with weight‐bearing as tolerated. From the sixth week, the patient wears a functional brace that allows a range of motion of 0° to full flexion. The brace is gradually discontinued after the third month.

## DISCUSSION

This technique supplements the technique previously published, which covered reconstruction of the sMCL with anteromedial reinforcement in cases of medial and anteromedial knee instability.^2^ The current technique presents a more extensive reconstruction, for cases in which posteromedial and posterior parts of the medial ligamentous complex are injured as well. Multiple authors have highlighted the importance of reconstruction individualization in accordance with which type of instability is present.^3^, ^14^, ^15^ For example, failure to address a posteromedial injury may result in persistent functional instability even if the sMCL has been reconstructed correctly.^16^, ^17^, ^18^, ^19^, ^20^ In 2025, Gawish et al. described a 3‐arm reconstruction of the dMCL, sMCL, and POL. However, with grafts pulled through 2 tibial tunnels, it does not allow for intraoperative isometry assessment and independent fixation of the grafts.^19^

In the technique, we describe a comprehensive 4‐arm method for reconstruction of the medial knee ligamentous complex from the most anterior to the most posterior part. The rationale for the first 2 arms of the technique was described in more detail in 2019.^2^ It aimed to address AMRI, AMI, and MI using a single autograft with minimum hardware and own blood supply. The importance of addressing AMRI, AMI, and MI to restore stability^21^, ^22^ and reduce the risk of anterior cruciate ligament reconstruction failure has been highlighted in multiple articles.^9^, ^10^, ^23^, ^24^ In particular, recent studies have shown the importance of adding the anteromedial graft to control AMRI, restoring the function of the dMCL.^25^, ^26^ Although the importance of the POL and PMC has been less extensively studied, they seem to be crucial for controlling internal rotation^27^, ^28^ and preventing excessive knee hyperextension.^29^, ^30^ Therefore, the posteromedial and posterior arms of the technique were introduced to recreate the functions of the POL and PMC, addressing PMI, PMRI, and PMAH. Importantly, it is crucial not to confuse PMAH with post‐traumatic lateral asymmetric hyperextension. These are relatively poorly defined types of instability, on which mainly cadaveric studies and surgical techniques have been published.^29^, ^30^ An important part of the technique is the intraoperative isometry assessment and independent graft fixation. As a result, it allows the range of patterns of instability to be addressed with no risk of overconstraint. The presented comprehensive medial knee 4‐arm reconstruction is aimed to address complex medial knee instability, using 2 hamstring autografts while minimizing tunnel convergence. It allows for intraoperative isometry assessment and independent graft tensioning and fixation.

## DISCLOSURES

The authors (K.M., K.M., M.B‐M., P.P., A.A.A., M.M.) declare that they have no known competing financial interests or personal relationships that could have appeared to influence the work reported in this article.
